# Does Regional Muscle Distribution Predict Functional Capacity? Sex-Specific Insights from Bioimpedance and Performance Testing

**DOI:** 10.3390/muscles5010017

**Published:** 2026-02-19

**Authors:** Elena Caso-Fontánez, Pablo López-Sierra, Carlos D. Gómez-Carmona, Sergio J. Ibáñez, Diego Muñoz

**Affiliations:** 1 Grupo de Optimización del Entrenamiento y el Rendimiento Deportivo (GOERD), Facultad de Ciencias del Deporte, Universidad de Extremadura, 10003 Cáceres, Spain; ecasofon@alumnos.unex.es (E.C.-F.); carlosdavid.gomez@unizar.es (C.D.G.-C.); sibanez@unex.es (S.J.I.); diegomun@unex.es (D.M.); 2 Research Group in Training, Physical Activity and Sports Performance (ENFYRED), University of Zaragoza, 50009 Zaragoza, Spain; 3 BioVetMed & SportSci Research Group, University of Murcia, 30100 Murcia, Spain

**Keywords:** body composition, handgrip strength, sexual dimorphism, core endurance, physically inactive occupations

## Abstract

Background: The relationships between segmental body composition and multidimensional performance outcomes remain insufficiently characterized in adults with limited but regular physical activity. This study examined associations between body composition parameters and functional test performance, while identifying sex-based differences. Methods: Forty-seven adults (31 women, 16 men; age 48.04 ± 11.33 years) underwent segmental bioelectrical impedance analysis and functional assessments including handgrip strength, isometric plank endurance, and single-leg balance. Correlations and sex comparisons statistical tests were performed. Results: Strong positive correlations were observed between segmental muscle mass and handgrip strength (r = 0.74–0.84, *p* < 0.05), with moderate associations for plank endurance (r = 0.30–0.32, *p* < 0.05). Fat mass demonstrated inverse relationships with performance, particularly for plank endurance (r = −0.36 to −0.62, *p* < 0.05). Males exhibited significantly greater muscle mass (*p* < 0.01), superior handgrip strength (*p* < 0.01), and longer plank times (*p* = 0.01). Females presented higher fat mass in lower limbs (*p* < 0.01). Conclusions: Segmental muscle mass shows strong associations with strength and moderate associations with core endurance, while adipose tissue exhibits inverse relationships. Pronounced sexual dimorphism exists in both body composition and functional capacity.

## 1. Introduction

Body composition assessment has emerged as a critical factor of metabolic health, functional capacity, and disease risk stratification. The precise quantification of skeletal muscle mass, adipose tissue distribution and bone mineral content provides relevant insights into cardiometabolic risk factors and functional performance capacity [1,2]. Bioelectrical Impedance Analysis (BIA) has gained acceptance as a non-invasive, cost-effective and reproducible methodology to assess whole-body and segmental body composition [3,4]. Recent multi-frequency BIA devices with eight-point tactile electrodes have demonstrated excellent validity and reliability compared to dual-energy X-ray absorptiometry (DXA) [5]. Segmental BIA enables region-specific assessment of tissue distribution. It facilitates the identification of asymmetries and localized deficits that may contribute to metabolic dysregulation or functional impairment [6]. Beyond descriptive applications, the evaluation of trunk and limb-specific muscle and fat mass may provide functionally relevant information when linked to distinct physical capacities, such as strength, core endurance, and postural control. However, such integrative approaches remain limited in adults with predominantly sedentary occupations who engage in regular but low-frequency exercise, highlighting the need for studies that jointly examine regional muscle and fat mass that may have significant implications for functional capacity and metabolic health [7].

Functional performance assessment constitutes an integral component of comprehensive health evaluation. It serves as a surrogate marker for independence, morbidity risk, and overall well-being across the lifespan. Handgrip strength (HGS) represents a well-established functional indicator of muscular strength and functional health [8,9]. HGS has demonstrated robust associations with all-cause mortality, cardiovascular disease incidence, and disability progression [9,10]. Core muscular endurance, quantified through isometric tests such as the prone plank, reflects the neuromuscular capacity to maintain trunk stability and has been implicated in lower back pain prevention and athletic performance [11,12]. Static postural control, assessed via single-leg stance protocols, provides critical information regarding sensorimotor integration, proprioceptive function, and fall risk, particularly relevant in sedentary populations [13]. The concurrent evaluation of body composition and multidimensional functional outcomes enables comprehensive phenotypic profiling to inform targeted exercise prescription and risk stratification.

Differences between sexes in body composition and functional performance have been extensively documented in the scientific literature [14]. Males typically present higher absolute and relative skeletal muscle mass, lower body fat percentage, and superior maximal force production compared to females. These differences are largely attributed to hormonal influences, particularly testosterone, as well as differences in habitual physical activity [15,16]. These sex-specific differences necessitate the development of normative reference values stratified by biological sex and underscore the importance of sex-disaggregated analyses in exercise physiology research. However, the magnitude of sexual dimorphism varies considerably as a function of age, physical activity level, nutritional status, and genetic predisposition. These results indicate the need for population-specific investigation [17].

Despite substantial progress in the analysis of body composition and functional capacity relationships, significant knowledge gaps persist. It regards the associations between segmental tissue distribution and domain-specific performance outcomes in adults with limited physical activity engagement. Existing literature has predominantly focused on elite athletic populations, clinical patient cohorts, or community-dwelling older adults [18,19]. In contrast, less attention has been directed to middle-aged individuals with sedentary occupations who participate in regular health-oriented exercise programs. Few investigations have simultaneously examined correlations between limb-specific muscle and fat mass indices and comprehensive functional assessments encompassing maximal strength, isometric endurance, and postural control. Clarifying these structure-function relationships in ecologically valid settings is necessary for optimizing assessment protocols, refining exercise prescription methodologies, and developing evidence-based intervention strategies [20].

Therefore, the present study aimed to examine the relationships between total and segmental body composition parameters derived from bioelectrical impedance analysis and performance outcomes in functional tests (handgrip strength, core muscular endurance, and static balance), as well as to identify sex-based differences in body composition profiles and functional performance in adults with physically inactive occupations who engage in regular but low-frequency structured exercise, representing a large proportion of the general population. It is hypothesized that: (a) segmental skeletal muscle mass will demonstrate significant positive correlations with strength and endurance performance, (b) adipose tissue mass will exhibit inverse associations with functional test outcomes, and (c) males will demonstrate greater skeletal muscle mass and higher functional performance, and lower relative adiposity compared to females.

## 2. Materials and Methods

### 2.1. Design

This research followed an ex post facto design, as it analyzed quantitative data retrospectively without experimental manipulation of variables [21]. The study was framed within a correlational and comparative approach, aiming to examine relationships and differences between selected variables based on naturally occurring characteristics of the participants. This non-experimental design allows for the identification of associations and group differences under real-world conditions, maintaining ecological validity within the context of applied sport and exercise sciences [22].

Sex was considered the main grouping of the study. The dependent variables included all body composition parameters obtained from the bioelectrical impedance analysis (total and segmental values of muscle mass, fat mass, bone mass, protein content, visceral fat index, and metabolic age), as well as the performance outcomes from the functional tests. These comprised the plank endurance time (seconds), handgrip strength (kg), and the balance performance assessed by the number of recorded errors.

### 2.2. Participants

A total of 47 adults, 31 women and 16 men, voluntarily participated in this study (Table 1). All participants were regular clients of a private sports clinic, where they attended two weekly training sessions focused primarily on functional strength exercises combined with moderate-intensity aerobic activities. Although all participants were considered healthy and free from medical conditions that could contraindicate exercise, most reported predominantly sedentary occupational habits, typically involving seated work in office-based environments. None of the participants engaged in additional structured physical activity outside of the supervised training sessions at the clinic.

Participants’ physical activity levels were assessed through a combination of direct monitoring and registration data. Attendance at the supervised training sessions was recorded by the investigators. Occupational physical activity was documented using a standardized registration questionnaire completed by participants at enrollment. Based on this information, participants were characterized as adults with predominantly physically inactive occupations who engaged in regular but low-frequency structured exercise.

All participants were informed about the objectives, potential benefits, and risks associated with the research. Written informed consent was obtained from all participants prior to data collection. The study was conducted in accordance with the principles outlined in the Declaration of Helsinki (2013) and approved by the University Bioethics Committee (protocol code 249/2025).

#### Eligibility Criteria

Participants were included if they were adults aged between 18 and 70 years, regularly attended two supervised training sessions per week at the clinic, and reported predominantly sedentary occupational routines outside the clinic sessions. All participants were generally healthy and had no medical contraindications to moderate-intensity exercise.

Exclusion criteria included the presence of acute or chronic conditions that could affect neuromuscular performance or balance, recent musculoskeletal injury, or any inability to safely complete the testing procedures (handgrip strength, plank endurance, and balance tests).

### 2.3. Measurements

Body composition was assessed using a segmental BIA device with four-point contact electrodes (hands and feet). The recorded variables included total body mass (kg), absolute and relative fat mass (kg and %), absolute and relative muscle mass (kg and %), lean mass (kg), absolute and relative bone mass (kg and %), absolute and relative protein mass (kg and %), visceral fat index (arbitrary units), and metabolic age (years). Metabolic age is a proprietary index provided by the bioelectrical impedance device, derived from internal algorithms, and is included solely as a descriptive parameter; it should not be interpreted as a clinical or physiological indicator of biological aging. Segmental analysis was performed to obtain absolute values of muscle mass and fat mass for the trunk, both upper limbs, and lower limbs. All measurements were taken following standardized pre-assessment conditions: participants refrained from alcohol and vigorous exercise for at least 24 h, avoided large meals within 4 h prior to testing, and voided their bladder 30–60 min before assessment.

The plank endurance test was used to assess core muscular endurance following the procedures described by [23,24]. Participants began in a prone position with elbows and toes in contact with the floor. The test started once both knees were lifted off the ground, maintaining only the forearms and toes as points of support. The forearms were aligned beneath the shoulders, elbows flexed at 90°, and hands placed shoulder-width apart with palms on the floor. The body was kept in a straight line from shoulders to ankles, avoiding hip elevation or sagging. Participants were instructed to maintain the position for as long as possible, and timing ceased upon any loss of position or when any part of the body (other than the forearms and toes) contacted the ground.

Static balance was evaluated using a 20 s single-leg stance test on the non-dominant leg, performed barefoot on a foam mat [25]. Participants stood with their contralateral (dominant) leg flexed without contacting the support leg, hands placed on the hips, and eyes closed. The test lasted a maximum of 20 s, during which the number of balance errors was recorded. Errors were defined as any of the following: touching the floor with the lifted foot, removing hands from the hips, excessive trunk or hip movement, or opening the eyes. A maximum of 10 errors could be scored. Participants unable to maintain the position for at least 5 s with eyes closed automatically received the maximum error score.

Maximal isometric handgrip strength was measured according to the standardized protocol of the Australian Hand Therapy Association (AHTA) [26]. Participants were seated upright with the hips and knees flexed at 90°, feet flat on the floor, and the tested arm positioned with the elbow flexed at 90°, the forearm in neutral rotation, and the wrist slightly extended (0–30°) and ulnarly deviated (0–15°). The non-tested arm remained relaxed alongside the body. Each participant performed two maximal trials with each hand, separated by 30 s of rest, and the best score was recorded for analysis.

### 2.4. Instruments

Body composition was assessed using a segmental bioelectrical impedance analyzer (Tanita MC-780MA, Tanita Corp., Tokyo, Japan), which provides multi-frequency analysis through eight tactile electrodes (hands and feet). This device has demonstrated high test–retest reliability and validity for estimating both total and segmental body composition in adults [27]. Data were automatically processed using the manufacturer’s software, and all measurements were conducted according to standardized operational procedures.

Maximal handgrip strength was measured using a digital hand dynamometer (Takei TKK 5401, Takei Scientific Instruments Co., Niigata, Japan), a device widely recognized for evaluating isometric grip strength in adults [28,29,30]. The dynamometer was calibrated before testing, and measurements were performed in accordance with the standardized protocol of the Australian Hand Therapy Association [26].

### 2.5. Procedure

The study was conducted in collaboration with a private sports clinic that provides functional and health-oriented exercise programs. The research team contacted the clinic’s coaches to propose an evaluation of selected physical and body composition variables. Following the coaches’ agreement, all participants were informed about the study procedures, and written informed consent was obtained prior to participation.

All assessments were performed over two non-consecutive days within the same week and were scheduled at the same time of day for each participant to minimize fatigue and circadian influences. The testing protocol included the evaluation of body composition through bioelectrical impedance analysis (BIA), an isometric plank endurance test, a single-leg balance test on the non-dominant leg, and a handgrip strength test.

On the first day, participants completed the body composition assessment using bioelectrical impedance analysis, followed by the isometric plank endurance test. On the second day, the single-leg balance test and the handgrip strength assessment were performed. All measurements were conducted under standardized environmental conditions at the same time of day for each participant to minimize circadian variation.

All testing sessions were supervised by two experienced evaluators previously trained in the study protocols to ensure procedural consistency and reliability. Data were immediately recorded in standardized templates and later exported to a secure database for statistical processing. Before analysis, all variables were reviewed to verify completeness and detect potential outliers or entry errors.

### 2.6. Statistical Analysis

All statistical analyses were performed using Jamovi (version 2.3.28; The Jamovi Project, Sydney, Australia). Descriptive statistics were calculated for all variables, including mean, standard deviation, minimum, and maximum values. Prior to inferential testing, assumption criteria were examined through tests of normality (Shapiro–Wilk) and homogeneity of variances (Levene’s test) to determine the suitability of parametric or non-parametric procedures.

To explore associations between body composition parameters and performance outcomes, Pearson’s product–moment correlation coefficients were calculated for normally distributed variables, and Spearman’s rank correlation coefficients were used for non-normally distributed data. To identify sex-related differences, independent samples *t*-tests were applied for parametric variables, while the Mann–Whitney U test was employed for non-parametric variables. Effect sizes were computed for each comparison: Cohen’s d for parametric tests and the rank biserial correlation (r_β_) for non-parametric tests.

The alpha level for statistical significance was set at *p* ≤ 0.05. Effect sizes for mean comparisons were interpreted as low (0–0.19), moderate (0.20–0.59), large (0.60–1.19), and very large (≥1.20) for Cohen’s d, and as trivial (0–0.09), small (0.10–0.29), moderate (0.30–0.49), large (0.50–0.69), and very large (≥0.70) for the rank biserial correlation [31]. All analyses were conducted with a confidence level of 95%.

## 3. Results

Table 2 presents the descriptive statistics for the total sample and stratified by sex.

On average, men showed higher values in body mass, skeletal muscle mass, and lean body components, while women presented greater total and relative fat mass. The mean metabolic age was similar between sexes, with slightly higher variability among women. In performance outcomes, men exhibited greater handgrip strength and longer plank endurance times, whereas balance scores were comparable between sexes, showing minimal dispersion in both groups.

Table 3 displays the correlation matrix showing the relationships between the body composition parameters and the performance test outcomes for the total sample. Darker green cells represent stronger positive or negative correlations, while lighter tones indicate weaker associations. White cells correspond to non-significant correlations (*p* > 0.05).

To address the large number of correlations, analyses were grouped into functionally relevant families according to the performance outcomes (handgrip strength, plank endurance, and balance), and *p*-values were adjusted within each family using the Benjamini–Hochberg false discovery rate (FDR) procedure (q = 0.05). Only associations that survived FDR correction were interpreted as robust. Values highlighted in red indicate correlations that did not remain significant after FDR adjustment. After correction, all associations between handgrip strength (right and left) and muscle mass variables (total and segmental) remained significant, whereas the association with absolute fat mass (kg) did not survive correction. For plank endurance, only the strongest associations, namely the negative associations with total and lower-limb fat mass and the positive association with total muscular mass, remained significant after FDR correction.

In general, strong positive correlations were observed among the different lean and muscular mass indices, while these variables showed strong inverse relationships with fat mass indicators. Specifically, greater muscle mass in both upper and lower limbs was moderately to strongly associated with higher handgrip strength values. Conversely, higher fat mass tended to correlate negatively with plank endurance time. Balance performance did not show significant relationships with any variable.

Table 4 displays the results of the between-sex comparison analyses, conducted using Student’s *t*-test or Mann–Whitney U test depending on the normality of the data.

Significant differences were observed in most body composition variables. Men presented higher values in skeletal muscle mass in both upper and lower limbs (all *p* < 0.01; large effect sizes). In contrast, women showed significantly higher total fat mass, particularly in the lower limbs (*p* < 0.01; large effects). Regarding performance variables, men achieved significantly greater handgrip strength (*p* < 0.01; large effect), whereas no significant differences were found in balance performance between sexes (*p* > 0.05). Plank endurance time tended to be higher in men (*p* = 0.01; large effect).

## 4. Discussion

The main objective of this study was to examine the relationships between segmental body composition parameters and the results of functional performance tests, as well as to explore sex differences in a sample of adults with moderate levels of physical activity. The findings support the initial hypotheses, revealing significant correlations between muscle mass and performance in strength tests, and between total muscle mass and isometric endurance, as well as clear sex-based differences in both body composition and functional performance. In this context, the value of segmental body composition assessment lies in its integration with functional testing, allowing a more nuanced interpretation of how regional muscle and fat mass relate to distinct physical capacities.

The results revealed greater muscle mass and superior performance in handgrip strength and plank endurance tests in men, whereas women displayed higher fat mass, particularly in the lower limbs. This finding aligns with existing scientific evidence on sexual dimorphism in the distribution of muscle and fat, which is mediated by hormonal and genetic factors [32]. The differences in body composition between sexes reflect the influence of testosterone in men, which promotes greater muscle development. Conversely, women tend to accumulate more fat, especially in the lower extremities [33], as observed in the fat mass results for the legs. Understanding these differences should be considered for trainers when designing personalized training programs, as the goal should not simply be to increase muscle mass and reduce body fat in a generalized manner, but rather to individualize objectives and training approaches based on each context and, specifically, each sex.

In the balance test, no significant correlations were found with body composition, suggesting that postural control under unstable conditions can be dependent to neuromuscular and sensory factors, such as proprioception, than to the amount of muscle or fat present in the body [1]. This finding is consistent with previous studies indicating that static balance depends on the nervous system’s ability to integrate sensory information, and that factors such as aging and the decline of proprioception negatively affect performance in these tasks [34,35]. In fact, proprioception has been shown to decrease with age, underscoring the importance of incorporating targeted training to improve stability and balance [36]. For this population, it is essential to include exercises that enhance proprioception and postural control, particularly in older adults or individuals at greater risk of falls.

The comparative analyses between men and women revealed significant differences in several body composition and functional performance variables. Significant differences were detected in plank endurance time and handgrip strength, with men presenting higher values in both measures. These findings likely may be influenced by their greater muscle mass, particularly in the torso and upper limbs, reinforcing the association between muscle mass and overall strength [37]. In contrast, significant differences were also observed in fat accumulation in the legs, with women presenting higher values, a pattern likely influenced by hormonal factors [38]. These differences align with the typical physiological profiles of each sex, suggesting that both men and women display distinct characteristics that should be considered when designing training programs. However, after correction for multiple comparisons, only the associations with total muscle mass and fat mass remained robust, suggesting that plank endurance reflects a global rather than segment-specific contribution of body composition. Nevertheless, these tendencies should be interpreted as general patterns; training approaches must ultimately be individualized according to each person and their specific context.

Despite the differences in body composition, the balance tests did not reveal significant differences between men and women. This result suggests that static balance performance does not depend as strongly on body composition as strength or endurance tests do. Although men have greater muscle mass, which could theoretically enhance postural control, and women carry more fat in the lower limbs, which could increase the load under conditions of imbalance, these differences do not appear to significantly influence performance in the balance test [1]. This reinforces the idea that balance seems to be more strongly associated with neuromuscular and sensory factors, such as proprioception, which are crucial for maintaining stability under challenging postural conditions [36]. The ability of the central nervous system to integrate and process sensory information in real time is more determinant for balance performance than variations in muscle or fat mass [39]. Thus, proprioception plays a key role in postural control, indicating that balance training should focus on improving sensory integration and responsiveness to perturbations, regardless of sex-based differences in body composition.

This study presents certain limitations, such as an unequal distribution of men and women, which may have affected the statistical power of some comparisons. Also, a sensitivity power analysis for a two-tailed bivariate correlation (α = 0.05) indicated that, with the available sample size (N = 47), the study had 90% power to detect correlations of approximately |r| ≥ 0.45. Therefore, smaller associations may not have been detected with adequate statistical power. Although a validated static balance test was used, its limited resolution and absence of a dynamic component may have reduced sensitivity to subtle associations between body composition and postural control. As a strength, the study included an adequate sample size for the analyses performed, and all assessments were conducted on two non-consecutive days, which helped reduce fatigue effects and improve the accuracy of the results. Furthermore, the inclusion and exclusion criteria used ensured a homogeneous sample and the participation of healthy adults without medical conditions that could interfere with testing, thereby increasing the internal validity of the findings. Future research should aim to address these limitations by ensuring a more balanced distribution of sexes to improve the statistical power of comparisons. It would also be beneficial to expand the sample to include individuals from a wider range of physical activity levels, encompassing both sedentary populations and highly trained individuals. In addition, longitudinal studies with more diverse samples should be conducted to examine the long-term effects of body composition on functional performance. Moreover, incorporating more dynamic functional tests would allow for a more comprehensive evaluation of muscle performance and postural control across different contexts.

## 5. Conclusions

The results demonstrate that segmental muscle mass is positively and significantly associated with performance in strength tests, whereas isometric endurance is associated primarily with total muscle mass and fat mass. Additionally, the differences observed between men and women confirmed the presence of sexual dimorphism in body composition, with men presenting higher muscle mass and superior strength performance, while women exhibited greater fat accumulation, particularly in the lower extremities. However, no significant sex differences were found in balance performance, suggesting that postural control in this context may be more strongly influenced by neuromuscular and sensory factors than by body composition, although this interpretation should be considered in light of the characteristics of the balance test employed.

The findings of this study highlight the relevance of muscle mass for functional capacity in healthy but sedentary adults, particularly in relation to regional strength performance. In this pre-pathological context, regular participation in structured exercise appears sufficient to support strength-related outcomes associated with segmental muscle mass. However, balance performance did not show robust associations with body composition, suggesting that postural control may not be similarly preserved in sedentary adults despite the absence of overt pathology. These results indicate that sedentary behavior may have a differentiated impact on functional domains, with balance potentially requiring specific proprioceptive and neuromuscular stimuli beyond those provided by low-frequency strength-oriented exercise. Accordingly, exercise interventions in sedentary but otherwise healthy adults should not only address muscular strength but also incorporate targeted balance and proprioceptive training, particularly to mitigate early functional alterations that may precede clinical impairment.

## 6. Practical Applications

Based on the results obtained in this study, trainers should consider the differences in segmental body composition between men and women when designing exercise programs, as these differences were associated with influence functional performance in strength and core endurance tests. Since greater segmental muscle mass, particularly in the upper limbs and trunk, was strongly associated with higher handgrip strength and total muscle mass with longer plank endurance, all individuals should incorporate resistance training that targets both upper- and lower-body muscle groups. In this sense, although men showed higher muscle mass in the arms and trunk, women, who on average presented lower upper-body muscle mass, may find particular benefit in emphasizing upper-body strengthening exercises such as rows, band pulls, or push-up variations, as these could help improve functional strength reflected in handgrip performance. Conversely, men, despite having higher muscle mass overall, may need to reinforce lower-body strength work to compensate for relative imbalances between upper-limb and lower-limb development, using exercises such as squats or lunges to enhance functional symmetry.

Because women exhibited significantly greater fat mass in the lower limbs, and fat mass showed negative associations with plank performance, particularly total and lower-limb fat mass, trainers can be incorporate strategies that progressively improve core endurance, such as isometric holds (e.g., planks or side planks), this can help counteract the additional load that higher fat mass may impose in isometric tasks. Importantly, these recommendations apply to both sexes, as reducing the relative demand placed on the trunk muscles through strength and endurance improvements is beneficial regardless of sex.

Balance and proprioceptive training represent another essential component. Since performance in the balance test was not associated with muscle mass or fat mass in any body segment, and did not differ between sexes, trainers should understand that improvements in balance appear to be more strongly related to on neuromuscular and sensory integration, rather than on changes in body composition. To enhance these abilities, exercises involving single-leg stands, eyes-closed balance tasks, or controlled movements on compliant surfaces (such as foam pads) can be incorporated progressively. These modalities may be particularly valuable for older adults or individuals with lower baseline balance scores.

Finally, integrating core-focused exercises is essential, given that plank endurance showed moderate positive associations with muscle mass and moderate negative associations with fat mass. Exercises such as side planks, hip lifts, and dynamic trunk stability drills should be progressed gradually to enhance both strength and postural control, contributing to improved functional capacity in daily tasks. Considering that this population engaged in only two weekly training sessions, trainers should prioritize exercises that efficiently target global strength and core endurance, as these were directly linked to better performance outcomes in this study.

These recommendations are particularly useful for practitioners working with adults who have predominantly sedentary occupations and engage in low-frequency structured exercise. Based on the present findings: (1) segmental muscle mass, especially in the upper limbs and trunk, emerges as a key correlate of functional strength and core endurance and should be prioritized in resistance training programs; (2) higher fat mass is negatively associated with plank performance, supporting the inclusion of progressive core endurance training to reduce mechanical demands during isometric tasks; (3) balance performance appears to be largely independent of body composition, indicating that improvements in postural control require specific proprioceptive and neuromuscular training; and (4) segmental body composition assessment may assist practitioners in developing more individualized and functionally relevant exercise prescriptions.

## Figures and Tables

**Table 1:** Descriptive Statistics of Sample.

Sample	Age (Years)	Body Mass (kg)
Total	48.04 ± 11.33	69.02 ± 12.77
Male	49.60 ± 12.32	79.70 ± 11.33
Female	47.30 ± 10.91	63.5 ± 9.67

**Table 2:** Descriptive statistics of body composition and performance variables for the total sample and by sex.

	All Subjects			Male			Female		
Variables	*Avg ± SD*	*Min*	*Max*	*Avg ± SD*	*Min*	*Max*	*Avg ± SD*	*Min*	*Max*
Diff Metabolic Age—Age (a.u.)	−6.38 ± 9.34	−16.00	16.00	−7.94 ± 8.15	−15.00	15.00	−5.58 ± 9.93	−16.00	16.00
Total Weight (Kg)	69.03 ± 12.77	47.20	114.10	79.72 ± 11.33	66.00	114.10	63.51 ± 9.67	47.20	88.30
Fat Mass (%)	27.80 ± 7.12	7.12	15.30	21.23 ± 4.68	15.30	33.30	31.20 ± 5.65	18.40	43.00
Muscular Mass (%)	40.87 ± 4.02	32.28	47.92	44.58 ± 2.65	37.77	47.92	38.95 ± 3.18	32.28	46.19
Proteins (%)	17.41 ± 2.01	13.50	22.70	19.49 ± 1.25	17.40	22.70	16.34 ± 1.39	13.50	19.90
Bone Tissue (%)	3.66 ± 0.35	2.80	4.20	3.94 ± 0.27	3.20	4.20	3.51 ± 0.30	2.80	4.20
Visceral Fat Index (a.u.)	6.75 ± 3.21	1.00	14.00	9.44 ± 3.25	4.00	14.00	5.36 ± 2.17	1.00	9.00
Torso Weight (Kg)	38.30 ± 7.36	25.30	62.20	45.18 ± 6.02	36.50	62.20	34.75 ± 5.17	25.30	48.40
Torso Fat Mass (Kg)	10.17 ± 3.71	2.50	22.60	10.76 ± 3.87	6.30	22.60	9.86 ± 3.64	2.50	20.20
Torso Muscular Mass (Kg)	26.79 ± 4.97	18.80	37.90	32.79 ± 2.79	28.20	37.90	23.69 ± 2.19	18.80	29.20
Left Arm Weight (Kg)	3.58 ± 0.94	0.94	2.20	4.43 ± 0.82	3.50	6.90	3.15 ± 0.68	2.20	5.10
Left Arm Fat Mass (Kg)	1.03 ± 0.47	0.40	2.60	0.98 ± 0.47	0.60	2.60	1.05 ± 0.47	0.40	2.40
Left Arm Muscular Mass (Kg)	2.42 ± 0.68	1.50	4.00	3.24 ± 0.45	2.50	4.00	2.00 ± 0.24	1.50	2.60
Right Arm Weight (Kg)	3.51 ± 0.90	2.30	6.80	4.39 ± 0.77	3.50	6.80	3.06 ± 0.57	2.30	4.70
Right Arm Fat Mass (Kg)	0.96 ± 0.42	0.40	2.40	0.93 ± 0.42	0.60	2.40	0.98 ± 0.43	0.40	2.20
Right Arm Muscular Mass (Kg)	2.41 ± 0.68	1.50	4.10	3.25 ± 0.41	2.60	4.10	1.98 ± 0.21	1.50	2.40
Left Leg Weight (Kg)	11.77 ± 1.97	8.60	19.00	12.91 ± 2.09	10.60	19.00	11.18 ± 1.65	8.60	15.70
Left Leg Fat Mass (Kg)	3.77 ± 2.07	1.50	14.40	3.05 ± 3.15	1.50	14.40	4.14 ± 1.08	2.60	6.80
Left Leg Muscular Mass (Kg)	7.88 ± 2.16	5.40	16.30	10.29 ± 2.00	7.90	16.30	6.64 ± 0.67	5.40	8.40
Right Leg Weight (Kg)	11.93 ± 1.92	8.80	19.20	13.00 ± 1.94	10.70	19.20	11.38 ± 1.69	8.80	16.00
Right Leg Fat Mass (Kg)	3.55 ± 1.37	1.40	6.80	2.31 ± 0.96	1.40	5.30	4.19 ± 1.09	2.80	6.80
Right Leg Muscular Mass (Kg)	7.92 ± 1.84	5.50	13.20	10.14 ± 1.20	7.90	13.20	6.78 ± 0.71	5.50	8.70
Plank (s)	127.32 ± 53.47	35.00	240.00	156.13 ± 43.49	90.00	240.00	112.45 ± 52.61	35.00	229.00
Balance non dominant leg (a.u.)	5.00 ± 3.34	0.00	10.00	5.13 ± 3.30	0.00	10.00	4.94 ± 3.42	0.00	10.00
Right-side Hand Dynamometer (Kg)	34.56 ± 8.71	22.30	56.80	45.10 ± 4.95	38.60	56.80	29.13 ± 3.78	22.30	37.10
Left-side Hand Dynamometer (Kg)	31.82 ± 9.08	21.30	62.30	42.18 ± 7.01	34.90	62.30	26.47 ± 3.88	21.30	35.70

Note: Avg: average; SD: standard deviation; min: minimum; max: maximum; a.u.: arbitrary units; kg: kilograms; %: percentage; s: seconds.

**Table 3:** Correlation coefficients between body composition and performance variables.

	Age	Met. Age	Plank	Balance	HG-R	HG-L	Weight	Fat M	Musc. M	Visceral Fat	T-FM	T-MM	L-A FM	L-A MM	R-A FM	R-A MM	L-L FM	L-L MM	R L FM		
**Met. Age**	0.67																				
**Plank**	−0.20	−0.46																			
**Balance**	0.41	0.32	−0.15																		
**HG Right**	−0.20	−0.25	0.44	0.08																	
**HG Left**	−0.20	−0.17	0.42	0.01	0.91																
**Weight**	0.03	0.43	0.00	0.06	0.54	0.61															
**Fat Mass**	0.01	0.54	−0.62	−0.06	−0.71	−0.62	−0.02														
**Muscular Mass**	−0.00	−0.54	0.62	0.06	0.71	0.62	0.02	−1.00													
**Visceral Fat**	0.56	0.77	−0.19	0.21	0.26	0.36	0.75	0.07	−0.07											1.00	Very large
**Torso FM**	0.24	0.77	−0.37	0.03	−0.04	0.08	0.75	0.55	−0.55	0.76										0.90	
**Torso MM**	−0.10	0.03	0.32	0.04	0.81	0.84	0.85	−0.46	0.46	0.53	0.34									0.80	
**L Arm FM**	0.05	0.67	−0.36	−0.08	−0.16	−0.03	0.64	0.68	−0.68	0.58	0.91	0.22								0.70	Large
**L Arm MM**	−0.10	0.11	0.25	0.07	0.76	0.81	0.89	−0.41	0.40	0.59	0.42	0.98	0.27							0.60	
**R Arm FM**	0.02	0.64	−0.40	−0.09	−0.13	−0.02	0.64	0.67	−0.67	0.57	0.91	0.21	0.98	0.27						0.50	Moderate
**R Arm MM**	−0.00	0.08	0.30	0.08	0.76	0.79	0.85	−0.46	0.46	0.56	0.37	0.97	0.20	0.99	0.20					0.40	
**L Leg FM**	−0.20	0.37	−0.49	−0.13	−0.52	−0.44	0.08	0.88	−0.87	0.06	0.51	−0.28	0.67	−0.26	0.65	−0.30				0.30	Small
**L Leg MM**	−0.10	0.07	0.31	0.05	0.74	0.76	0.87	−0.43	0.42	0.56	0.41	0.95	0.26	0.96	0.26	0.95	−0.29			0.20	
**R Leg FM**	−0.10	0.46	−0.55	−0.11	−0.62	−0.54	0.01	0.94	−0.94	−0.02	0.48	−0.37	0.66	−0.34	0.65	−0.39	0.92	−0.39		0.10	Trivial
**R Leg MM**	−0.10	0.03	0.32	0.06	0.76	0.77	0.84	−0.44	0.44	0.51	0.37	0.95	0.30	0.95	0.23	0.95	−0.32	0.99	−0.40	0.00	

Note: The green color gradient represents the correlation coefficient. All cells shaded in green indicate statistically significant correlations (*p* < 0.05). Greater color intensity corresponds to stronger correlations, whereas lower color intensity corresponds to weaker correlations. The interpretation of the green color scale is provided on the right-hand side of the table.

**Table 4:** Results of between-sex comparison analyses for body composition and performance variables.

Variable	Test	Statistic	*p*-Value	ES	Differences
Plank (s)	t	2.85	**0.01**	0.88	Male
Balance nDL (a.u.)	U	236.50	0.80	−0.05	
HG Right (Kg)	U	0.00	**<0.01**	−1.00	Male
HR Left (Kg)	U	1.50	**<0.01**	−0.99	Male
Torso Fat Mass (Kg)	U	205.50	0.35	−0.17	
Torso Muscular Mass (Kg)	U	2.00	**<0.01**	−0.99	Male
Left Arm Fat Mass (Kg)	U	231.50	0.72	0.07	
Left Arm Muscular Mass (Kg)	U	2.00	**<0.01**	−0.99	Male
Right Arm Fat Mass (Kg)	U	235.00	0.78	0.05	
Right Arm Muscular Mass (Kg)	U	0.00	**<0.01**	−1.00	Male
Left Leg Fat Mass (Kg)	U	62.00	**<0.01**	0.75	Female
Left Leg Muscular Mass (Kg)	U	2.00	**<0.01**	−0.99	Male
Right Leg Fat Mass (Kg)	t	−5.83	**<0.01**	3.72	Female
Right Leg Muscular Mass (Kg)	U	3.50	**<0.01**	−0.99	Male

Note: ES: effect size; a.u.: arbitrary units; kg: kilograms; s: seconds. nDL = Non-Dominant Leg, HG = Hand Grip. Values in bold indicate statistically significant results (*p* < 0.05).

## Data Availability

Data are unavailable due to privacy restrictions.
